# Supplementary material for: Pyruvate Dehydrogenase Kinase 1 inhibition mediated oxidative phosphorylation enhancement in cartilage promotes osteoarthritis progression
Source: BMC Musculoskelet Disord. 2023 Jul 20;24:597. doi: 10.1186/s12891-023-06585-6 (PMC10357736; doi:10.1186/s12891-023-06585-6)
Supplement: Supplementary file 1 — Additional file 1. [file 12891_2023_6585_MOESM1_ESM.zip › KEGG.Gsea.1653622432464/KEGG_JAK_STAT_SIGNALING_PATHWAY.html]

Details for gene set KEGG\_JAK\_STAT\_SIGNALING\_PATHWAY[GSEA]

|  || Dataset | 55457\_collapsed\_to\_symbols.cls.cls #OA\_versus\_normal.cls.cls #OA\_versus\_normal\_repos |
| Phenotype | cls.cls#OA\_versus\_normal\_repos |
| Upregulated in class | normal |
| GeneSet | KEGG\_JAK\_STAT\_SIGNALING\_PATHWAY |
| Enrichment Score (ES) | -0.3534063 |
| Normalized Enrichment Score (NES) | -1.6132882 |
| Nominal p-value | 0.001529052 |
| FDR q-value | 0.14727773 |
| FWER p-Value | 0.62 |
Table: GSEA Results Summary

  

Fig 1: Enrichment plot: KEGG\_JAK\_STAT\_SIGNALING\_PATHWAY      
 Profile of the Running ES Score & Positions of GeneSet Members on the Rank Ordered List

  

| SYMBOL | TITLE | RANK IN GENE LIST | RANK METRIC SCORE | RUNNING ES | CORE ENRICHMENT || 1 | IL23A | interleukin 23 subunit alpha [Source:HGNC Symbol;Acc:HGNC:15488] | 312 | 0.740 | -0.0064 | No |
| 2 | IL11RA | interleukin 11 receptor subunit alpha [Source:HGNC Symbol;Acc:HGNC:5967] | 500 | 0.659 | -0.0049 | No |
| 3 | IL10RA | interleukin 10 receptor subunit alpha [Source:HGNC Symbol;Acc:HGNC:5964] | 588 | 0.628 | 0.0036 | No |
| 4 | IFNAR2 | interferon alpha and beta receptor subunit 2 [Source:HGNC Symbol;Acc:HGNC:5433] | 616 | 0.618 | 0.0165 | No |
| 5 | IL20RA | interleukin 20 receptor subunit alpha [Source:HGNC Symbol;Acc:HGNC:6003] | 668 | 0.601 | 0.0272 | No |
| 6 | IFNGR2 | interferon gamma receptor 2 [Source:HGNC Symbol;Acc:HGNC:5440] | 861 | 0.552 | 0.0256 | No |
| 7 | AKT1 | AKT serine/threonine kinase 1 [Source:HGNC Symbol;Acc:HGNC:391] | 1192 | 0.478 | 0.0114 | No |
| 8 | IL21 | interleukin 21 [Source:HGNC Symbol;Acc:HGNC:6005] | 1199 | 0.477 | 0.0226 | No |
| 9 | IL13RA2 | interleukin 13 receptor subunit alpha 2 [Source:HGNC Symbol;Acc:HGNC:5975] | 1202 | 0.476 | 0.0341 | No |
| 10 | IFNA8 | interferon alpha 8 [Source:HGNC Symbol;Acc:HGNC:5429] | 1206 | 0.475 | 0.0454 | No |
| 11 | SOCS5 | suppressor of cytokine signaling 5 [Source:HGNC Symbol;Acc:HGNC:16852] | 1543 | 0.421 | 0.0294 | No |
| 12 | IRF9 | interferon regulatory factor 9 [Source:HGNC Symbol;Acc:HGNC:6131] | 1748 | 0.392 | 0.0230 | No |
| 13 | IL12RB2 | interleukin 12 receptor subunit beta 2 [Source:HGNC Symbol;Acc:HGNC:5972] | 1857 | 0.379 | 0.0238 | No |
| 14 | PIK3CB | "phosphatidylinositol-4,5-bisphosphate 3-kinase catalytic subunit beta [Source:HGNC Symbol;Acc:HGNC:8976]" | 2186 | 0.340 | 0.0064 | No |
| 15 | IL7 | interleukin 7 [Source:HGNC Symbol;Acc:HGNC:6023] | 2320 | 0.324 | 0.0038 | No |
| 16 | EPO | erythropoietin [Source:HGNC Symbol;Acc:HGNC:3415] | 2369 | 0.318 | 0.0078 | No |
| 17 | IL10RB | interleukin 10 receptor subunit beta [Source:HGNC Symbol;Acc:HGNC:5965] | 2438 | 0.309 | 0.0101 | No |
| 18 | GH1 | growth hormone 1 [Source:HGNC Symbol;Acc:HGNC:4261] | 2456 | 0.308 | 0.0162 | No |
| 19 | CCND2 | cyclin D2 [Source:HGNC Symbol;Acc:HGNC:1583] | 2508 | 0.302 | 0.0196 | No |
| 20 | IL15 | interleukin 15 [Source:HGNC Symbol;Acc:HGNC:5977] | 2599 | 0.291 | 0.0197 | No |
| 21 | PTPN6 | protein tyrosine phosphatase non-receptor type 6 [Source:HGNC Symbol;Acc:HGNC:9658] | 3434 | 0.208 | -0.0406 | No |
| 22 | IFNAR1 | interferon alpha and beta receptor subunit 1 [Source:HGNC Symbol;Acc:HGNC:5432] | 3587 | 0.195 | -0.0477 | No |
| 23 | CSH1 | chorionic somatomammotropin hormone 1 [Source:HGNC Symbol;Acc:HGNC:2440] | 3634 | 0.191 | -0.0467 | No |
| 24 | CSF2 | colony stimulating factor 2 [Source:HGNC Symbol;Acc:HGNC:2434] | 3699 | 0.185 | -0.0472 | No |
| 25 | CSF2RA | colony stimulating factor 2 receptor subunit alpha [Source:HGNC Symbol;Acc:HGNC:2435] | 3797 | 0.178 | -0.0504 | No |
| 26 | CTF1 | cardiotrophin 1 [Source:HGNC Symbol;Acc:HGNC:2499] | 4064 | 0.150 | -0.0676 | No |
| 27 | IL9R | interleukin 9 receptor [Source:HGNC Symbol;Acc:HGNC:6030] | 4118 | 0.144 | -0.0683 | No |
| 28 | JAK2 | Janus kinase 2 [Source:HGNC Symbol;Acc:HGNC:6192] | 4183 | 0.140 | -0.0699 | No |
| 29 | IFNA4 | interferon alpha 4 [Source:HGNC Symbol;Acc:HGNC:5425] | 4282 | 0.129 | -0.0744 | No |
| 30 | IL2RA | interleukin 2 receptor subunit alpha [Source:HGNC Symbol;Acc:HGNC:6008] | 4292 | 0.129 | -0.0720 | No |
| 31 | PRLR | prolactin receptor [Source:HGNC Symbol;Acc:HGNC:9446] | 4323 | 0.126 | -0.0712 | No |
| 32 | IL11 | interleukin 11 [Source:HGNC Symbol;Acc:HGNC:5966] | 4327 | 0.126 | -0.0684 | No |
| 33 | SOCS7 | suppressor of cytokine signaling 7 [Source:HGNC Symbol;Acc:HGNC:29846] | 4337 | 0.125 | -0.0660 | No |
| 34 | CCND3 | cyclin D3 [Source:HGNC Symbol;Acc:HGNC:1585] | 4426 | 0.119 | -0.0700 | No |
| 35 | STAT1 | signal transducer and activator of transcription 1 [Source:HGNC Symbol;Acc:HGNC:11362] | 4495 | 0.113 | -0.0726 | No |
| 36 | EPOR | erythropoietin receptor [Source:HGNC Symbol;Acc:HGNC:3416] | 4596 | 0.105 | -0.0779 | No |
| 37 | IL21R | interleukin 21 receptor [Source:HGNC Symbol;Acc:HGNC:6006] | 4810 | 0.087 | -0.0924 | No |
| 38 | IL2RG | interleukin 2 receptor subunit gamma [Source:HGNC Symbol;Acc:HGNC:6010] | 4887 | 0.081 | -0.0964 | No |
| 39 | CSF3 | colony stimulating factor 3 [Source:HGNC Symbol;Acc:HGNC:2438] | 4995 | 0.073 | -0.1030 | No |
| 40 | IL2RB | interleukin 2 receptor subunit beta [Source:HGNC Symbol;Acc:HGNC:6009] | 5045 | 0.069 | -0.1052 | No |
| 41 | CNTF | ciliary neurotrophic factor [Source:HGNC Symbol;Acc:HGNC:2169] | 5165 | 0.059 | -0.1130 | No |
| 42 | SPRED2 | sprouty related EVH1 domain containing 2 [Source:HGNC Symbol;Acc:HGNC:17722] | 5179 | 0.058 | -0.1126 | No |
| 43 | PIAS3 | protein inhibitor of activated STAT 3 [Source:HGNC Symbol;Acc:HGNC:16861] | 5195 | 0.057 | -0.1124 | No |
| 44 | IL5 | interleukin 5 [Source:HGNC Symbol;Acc:HGNC:6016] | 5277 | 0.050 | -0.1176 | No |
| 45 | PRL | prolactin [Source:HGNC Symbol;Acc:HGNC:9445] | 5490 | 0.033 | -0.1334 | No |
| 46 | MPL | "MPL proto-oncogene, thrombopoietin receptor [Source:HGNC Symbol;Acc:HGNC:7217]" | 5508 | 0.032 | -0.1339 | No |
| 47 | IL24 | interleukin 24 [Source:HGNC Symbol;Acc:HGNC:11346] | 5517 | 0.031 | -0.1338 | No |
| 48 | STAT5B | signal transducer and activator of transcription 5B [Source:HGNC Symbol;Acc:HGNC:11367] | 5554 | 0.028 | -0.1359 | No |
| 49 | STAT4 | signal transducer and activator of transcription 4 [Source:HGNC Symbol;Acc:HGNC:11365] | 5584 | 0.025 | -0.1376 | No |
| 50 | GRB2 | growth factor receptor bound protein 2 [Source:HGNC Symbol;Acc:HGNC:4566] | 5697 | 0.016 | -0.1460 | No |
| 51 | IL2 | interleukin 2 [Source:HGNC Symbol;Acc:HGNC:6001] | 5819 | 0.007 | -0.1553 | No |
| 52 | IFNA2 | interferon alpha 2 [Source:HGNC Symbol;Acc:HGNC:5423] | 5869 | 0.004 | -0.1590 | No |
| 53 | IL12B | interleukin 12B [Source:HGNC Symbol;Acc:HGNC:5970] | 5991 | -0.004 | -0.1684 | No |
| 54 | IFNA21 | interferon alpha 21 [Source:HGNC Symbol;Acc:HGNC:5424] | 6127 | -0.015 | -0.1786 | No |
| 55 | STAM | signal transducing adaptor molecule [Source:HGNC Symbol;Acc:HGNC:11357] | 6403 | -0.035 | -0.1993 | No |
| 56 | IFNG | interferon gamma [Source:HGNC Symbol;Acc:HGNC:5438] | 6410 | -0.036 | -0.1989 | No |
| 57 | IL26 | interleukin 26 [Source:HGNC Symbol;Acc:HGNC:17119] | 6419 | -0.037 | -0.1986 | No |
| 58 | TPO | thyroid peroxidase [Source:HGNC Symbol;Acc:HGNC:12015] | 6467 | -0.040 | -0.2013 | No |
| 59 | IL4 | interleukin 4 [Source:HGNC Symbol;Acc:HGNC:6014] | 6673 | -0.054 | -0.2161 | No |
| 60 | IFNA10 | interferon alpha 10 [Source:HGNC Symbol;Acc:HGNC:5418] | 6682 | -0.054 | -0.2154 | No |
| 61 | BCL2L1 | BCL2 like 1 [Source:HGNC Symbol;Acc:HGNC:992] | 6698 | -0.055 | -0.2152 | No |
| 62 | GHR | growth hormone receptor [Source:HGNC Symbol;Acc:HGNC:4263] | 6740 | -0.058 | -0.2170 | No |
| 63 | IFNA5 | interferon alpha 5 [Source:HGNC Symbol;Acc:HGNC:5426] | 6755 | -0.059 | -0.2166 | No |
| 64 | IL12A | interleukin 12A [Source:HGNC Symbol;Acc:HGNC:5969] | 6930 | -0.074 | -0.2284 | No |
| 65 | STAT5A | signal transducer and activator of transcription 5A [Source:HGNC Symbol;Acc:HGNC:11366] | 6988 | -0.079 | -0.2310 | No |
| 66 | GH2 | growth hormone 2 [Source:HGNC Symbol;Acc:HGNC:4262] | 6993 | -0.079 | -0.2293 | No |
| 67 | JAK1 | Janus kinase 1 [Source:HGNC Symbol;Acc:HGNC:6190] | 6995 | -0.079 | -0.2275 | No |
| 68 | IFNGR1 | interferon gamma receptor 1 [Source:HGNC Symbol;Acc:HGNC:5439] | 7176 | -0.094 | -0.2393 | No |
| 69 | CRLF2 | cytokine receptor like factor 2 [Source:HGNC Symbol;Acc:HGNC:14281] | 7254 | -0.100 | -0.2429 | No |
| 70 | IFNA6 | interferon alpha 6 [Source:HGNC Symbol;Acc:HGNC:5427] | 7272 | -0.101 | -0.2418 | No |
| 71 | PIK3CD | "phosphatidylinositol-4,5-bisphosphate 3-kinase catalytic subunit delta [Source:HGNC Symbol;Acc:HGNC:8977]" | 7405 | -0.111 | -0.2494 | No |
| 72 | CCND1 | cyclin D1 [Source:HGNC Symbol;Acc:HGNC:1582] | 7512 | -0.119 | -0.2548 | No |
| 73 | LIFR | LIF receptor subunit alpha [Source:HGNC Symbol;Acc:HGNC:6597] | 7625 | -0.129 | -0.2604 | No |
| 74 | PIK3CA | "phosphatidylinositol-4,5-bisphosphate 3-kinase catalytic subunit alpha [Source:HGNC Symbol;Acc:HGNC:8975]" | 7733 | -0.136 | -0.2655 | No |
| 75 | CSF2RB | colony stimulating factor 2 receptor subunit beta [Source:HGNC Symbol;Acc:HGNC:2436] | 7826 | -0.143 | -0.2692 | No |
| 76 | PIK3CG | "phosphatidylinositol-4,5-bisphosphate 3-kinase catalytic subunit gamma [Source:HGNC Symbol;Acc:HGNC:8978]" | 7953 | -0.152 | -0.2754 | No |
| 77 | IL22 | interleukin 22 [Source:HGNC Symbol;Acc:HGNC:14900] | 7997 | -0.154 | -0.2750 | No |
| 78 | AKT3 | AKT serine/threonine kinase 3 [Source:HGNC Symbol;Acc:HGNC:393] | 8008 | -0.156 | -0.2720 | No |
| 79 | SOS2 | SOS Ras/Rho guanine nucleotide exchange factor 2 [Source:HGNC Symbol;Acc:HGNC:11188] | 8058 | -0.160 | -0.2719 | No |
| 80 | IL13RA1 | interleukin 13 receptor subunit alpha 1 [Source:HGNC Symbol;Acc:HGNC:5974] | 8085 | -0.162 | -0.2700 | No |
| 81 | IL22RA1 | interleukin 22 receptor subunit alpha 1 [Source:HGNC Symbol;Acc:HGNC:13700] | 8101 | -0.163 | -0.2672 | No |
| 82 | STAT6 | signal transducer and activator of transcription 6 [Source:HGNC Symbol;Acc:HGNC:11368] | 8191 | -0.170 | -0.2700 | No |
| 83 | STAT3 | signal transducer and activator of transcription 3 [Source:HGNC Symbol;Acc:HGNC:11364] | 8453 | -0.190 | -0.2858 | No |
| 84 | LEPR | leptin receptor [Source:HGNC Symbol;Acc:HGNC:6554] | 8479 | -0.192 | -0.2831 | No |
| 85 | IFNA7 | interferon alpha 7 [Source:HGNC Symbol;Acc:HGNC:5428] | 8533 | -0.196 | -0.2824 | No |
| 86 | IFNA1 | interferon alpha 1 [Source:HGNC Symbol;Acc:HGNC:5417] | 8640 | -0.204 | -0.2858 | No |
| 87 | LEP | leptin [Source:HGNC Symbol;Acc:HGNC:6553] | 8740 | -0.212 | -0.2884 | No |
| 88 | IL12RB1 | interleukin 12 receptor subunit beta 1 [Source:HGNC Symbol;Acc:HGNC:5971] | 8768 | -0.213 | -0.2853 | No |
| 89 | PIK3R3 | phosphoinositide-3-kinase regulatory subunit 3 [Source:HGNC Symbol;Acc:HGNC:8981] | 8801 | -0.217 | -0.2825 | No |
| 90 | PIK3R5 | phosphoinositide-3-kinase regulatory subunit 5 [Source:HGNC Symbol;Acc:HGNC:30035] | 8804 | -0.217 | -0.2773 | No |
| 91 | IL10 | interleukin 10 [Source:HGNC Symbol;Acc:HGNC:5962] | 8855 | -0.221 | -0.2759 | No |
| 92 | IL5RA | interleukin 5 receptor subunit alpha [Source:HGNC Symbol;Acc:HGNC:6017] | 9204 | -0.248 | -0.2971 | No |
| 93 | IL7R | interleukin 7 receptor [Source:HGNC Symbol;Acc:HGNC:6024] | 9233 | -0.251 | -0.2931 | No |
| 94 | CSF3R | colony stimulating factor 3 receptor [Source:HGNC Symbol;Acc:HGNC:2439] | 9247 | -0.252 | -0.2880 | No |
| 95 | CBL | Cbl proto-oncogene [Source:HGNC Symbol;Acc:HGNC:1541] | 9479 | -0.272 | -0.2995 | No |
| 96 | SPRY2 | sprouty RTK signaling antagonist 2 [Source:HGNC Symbol;Acc:HGNC:11270] | 9531 | -0.277 | -0.2967 | No |
| 97 | CBLC | Cbl proto-oncogene C [Source:HGNC Symbol;Acc:HGNC:15961] | 9553 | -0.279 | -0.2915 | No |
| 98 | SOCS2 | suppressor of cytokine signaling 2 [Source:HGNC Symbol;Acc:HGNC:19382] | 9802 | -0.302 | -0.3036 | No |
| 99 | IL3 | interleukin 3 [Source:HGNC Symbol;Acc:HGNC:6011] | 9850 | -0.306 | -0.2998 | No |
| 100 | CISH | cytokine inducible SH2 containing protein [Source:HGNC Symbol;Acc:HGNC:1984] | 10535 | -0.377 | -0.3442 | Yes |
| 101 | OSMR | oncostatin M receptor [Source:HGNC Symbol;Acc:HGNC:8507] | 10560 | -0.379 | -0.3368 | Yes |
| 102 | IL15RA | interleukin 15 receptor subunit alpha [Source:HGNC Symbol;Acc:HGNC:5978] | 10586 | -0.382 | -0.3295 | Yes |
| 103 | TYK2 | tyrosine kinase 2 [Source:HGNC Symbol;Acc:HGNC:12440] | 10756 | -0.399 | -0.3330 | Yes |
| 104 | IFNB1 | interferon beta 1 [Source:HGNC Symbol;Acc:HGNC:5434] | 10801 | -0.406 | -0.3265 | Yes |
| 105 | IFNW1 | interferon omega 1 [Source:HGNC Symbol;Acc:HGNC:5448] | 10816 | -0.407 | -0.3177 | Yes |
| 106 | IL19 | interleukin 19 [Source:HGNC Symbol;Acc:HGNC:5990] | 10846 | -0.410 | -0.3099 | Yes |
| 107 | CREBBP | CREB binding protein [Source:HGNC Symbol;Acc:HGNC:2348] | 10881 | -0.414 | -0.3025 | Yes |
| 108 | IL4R | interleukin 4 receptor [Source:HGNC Symbol;Acc:HGNC:6015] | 10902 | -0.417 | -0.2939 | Yes |
| 109 | JAK3 | Janus kinase 3 [Source:HGNC Symbol;Acc:HGNC:6193] | 10908 | -0.418 | -0.2841 | Yes |
| 110 | PIK3R2 | phosphoinositide-3-kinase regulatory subunit 2 [Source:HGNC Symbol;Acc:HGNC:8980] | 10956 | -0.425 | -0.2774 | Yes |
| 111 | PIK3R1 | phosphoinositide-3-kinase regulatory subunit 1 [Source:HGNC Symbol;Acc:HGNC:8979] | 11099 | -0.443 | -0.2777 | Yes |
| 112 | STAM2 | signal transducing adaptor molecule 2 [Source:HGNC Symbol;Acc:HGNC:11358] | 11194 | -0.456 | -0.2739 | Yes |
| 113 | CNTFR | ciliary neurotrophic factor receptor [Source:HGNC Symbol;Acc:HGNC:2170] | 11203 | -0.457 | -0.2634 | Yes |
| 114 | PTPN11 | protein tyrosine phosphatase non-receptor type 11 [Source:HGNC Symbol;Acc:HGNC:9644] | 11260 | -0.465 | -0.2564 | Yes |
| 115 | PIAS2 | protein inhibitor of activated STAT 2 [Source:HGNC Symbol;Acc:HGNC:17311] | 11288 | -0.470 | -0.2470 | Yes |
| 116 | IL9 | interleukin 9 [Source:HGNC Symbol;Acc:HGNC:6029] | 11309 | -0.472 | -0.2371 | Yes |
| 117 | PIAS1 | protein inhibitor of activated STAT 1 [Source:HGNC Symbol;Acc:HGNC:2752] | 11422 | -0.490 | -0.2339 | Yes |
| 118 | IL6R | interleukin 6 receptor [Source:HGNC Symbol;Acc:HGNC:6019] | 11615 | -0.519 | -0.2363 | Yes |
| 119 | EP300 | E1A binding protein p300 [Source:HGNC Symbol;Acc:HGNC:3373] | 11741 | -0.540 | -0.2329 | Yes |
| 120 | SOS1 | SOS Ras/Rac guanine nucleotide exchange factor 1 [Source:HGNC Symbol;Acc:HGNC:11187] | 12041 | -0.603 | -0.2416 | Yes |
| 121 | PIAS4 | protein inhibitor of activated STAT 4 [Source:HGNC Symbol;Acc:HGNC:17002] | 12060 | -0.610 | -0.2281 | Yes |
| 122 | LIF | LIF interleukin 6 family cytokine [Source:HGNC Symbol;Acc:HGNC:6596] | 12085 | -0.615 | -0.2150 | Yes |
| 123 | IL13 | interleukin 13 [Source:HGNC Symbol;Acc:HGNC:5973] | 12173 | -0.636 | -0.2063 | Yes |
| 124 | STAT2 | signal transducer and activator of transcription 2 [Source:HGNC Symbol;Acc:HGNC:11363] | 12196 | -0.644 | -0.1923 | Yes |
| 125 | CLCF1 | cardiotrophin like cytokine factor 1 [Source:HGNC Symbol;Acc:HGNC:17412] | 12360 | -0.695 | -0.1881 | Yes |
| 126 | IL3RA | interleukin 3 receptor subunit alpha [Source:HGNC Symbol;Acc:HGNC:6012] | 12380 | -0.703 | -0.1724 | Yes |
| 127 | SPRY4 | sprouty RTK signaling antagonist 4 [Source:HGNC Symbol;Acc:HGNC:15533] | 12441 | -0.721 | -0.1595 | Yes |
| 128 | SOCS3 | suppressor of cytokine signaling 3 [Source:HGNC Symbol;Acc:HGNC:19391] | 12453 | -0.725 | -0.1427 | Yes |
| 129 | IL6 | interleukin 6 [Source:HGNC Symbol;Acc:HGNC:6018] | 12460 | -0.730 | -0.1253 | Yes |
| 130 | PIM1 | "Pim-1 proto-oncogene, serine/threonine kinase [Source:HGNC Symbol;Acc:HGNC:8986]" | 12500 | -0.748 | -0.1101 | Yes |
| 131 | IL6ST | interleukin 6 cytokine family signal transducer [Source:HGNC Symbol;Acc:HGNC:6021] | 12505 | -0.749 | -0.0922 | Yes |
| 132 | SOCS1 | suppressor of cytokine signaling 1 [Source:HGNC Symbol;Acc:HGNC:19383] | 12664 | -0.836 | -0.0841 | Yes |
| 133 | CBLB | Cbl proto-oncogene B [Source:HGNC Symbol;Acc:HGNC:1542] | 12725 | -0.887 | -0.0672 | Yes |
| 134 | SPRY1 | sprouty RTK signaling antagonist 1 [Source:HGNC Symbol;Acc:HGNC:11269] | 12773 | -0.931 | -0.0481 | Yes |
| 135 | MYC | "MYC proto-oncogene, bHLH transcription factor [Source:HGNC Symbol;Acc:HGNC:7553]" | 12801 | -0.970 | -0.0266 | Yes |
| 136 | AKT2 | AKT serine/threonine kinase 2 [Source:HGNC Symbol;Acc:HGNC:392] | 12896 | -1.404 | 0.0003 | Yes |
Table: GSEA details [plain text format]

  

Fig 2: KEGG\_JAK\_STAT\_SIGNALING\_PATHWAY      
 Blue-Pink O' Gram in the Space of the Analyzed GeneSet

  

Fig 3: KEGG\_JAK\_STAT\_SIGNALING\_PATHWAY: Random ES distribution      
 Gene set null distribution of ES for **KEGG\_JAK\_STAT\_SIGNALING\_PATHWAY**
